# Use of p53 immunohistochemistry can improve diagnostic agreement for differentiated vulvar intraepithelial neoplasia (dVIN): an international reproducibility study

**DOI:** 10.1111/his.15524

**Published:** 2025-08-05

**Authors:** Shatavisha Dasgupta, Anne‐Sophie Van Rompuy, Christine Bergeron, Debra S Heller, Demaretta Rush, Francoise Plantier, James Scurry, Jennifer Roberts, Joost Bart, Kathleen Lambein, Katy Veprauskas, Koen K Van de Vijver, Lex ACF Makkus, Loes Kooreman, Maaike Bleeker, Mahfooz Basha Mohamed, Maria Angelica Selim, Maria Carmen Rodriguez Alvarez, Mieke R Van Bockstal, Noel Jean‐Christophe, Patricia Guzman, Radhika Srinivasan, Rupali Arora, Russell Ball, Suzanne Wilhelmus, Senada Koljenović, Folkert J van Kemenade, Patricia C Ewing‐Graham

**Affiliations:** ^1^ Department of Pathology Erasmus MC, University Medical Center Rotterdam Rotterdam The Netherlands; ^2^ Imaging Platform, Broad Institute of MIT and Harvard Cambridge Massachusetts USA; ^3^ Department of Pathology University Hospitals Leuven, KU Leuven Leuven Belgium; ^4^ Department of Pathology CerbaPath Paris France; ^5^ Department of Pathology, Immunology, & Laboratory Medicine Rutgers‐New Jersey Medical School Newark New Jersey USA; ^6^ Department of Pathology University of Arizona College of Medicine Tucson Arizona USA; ^7^ Department of Pathology Hôpital Cochin Paris France; ^8^ Department of Anatomical Pathology, NSW Health Pathology John Hunter Hospital Newcastle New South Wales Australia; ^9^ Department of Health and Medicine University of Newcastle Newcastle New South Wales Australia; ^10^ GynaePath, Douglass Hanly Moir Pathology Sydney New South Wales Australia; ^11^ Department of Pathology University Medical Center Groningen Groningen The Netherlands; ^12^ Department of Pathology AZ St Lucas Ghent Belgium; ^13^ Department of Surgical Oncology University Hospitals Leuven, KU Leuven Leuven Belgium; ^14^ Department of Pathology Pathology Specialists of New England Manchester New Hampshire USA; ^15^ Department of Pathology, Cancer Research Institute Ghent Ghent University Hospital Ghent Belgium; ^16^ Department of Pathology Antwerp University Antwerp Belgium; ^17^ Laboratory for Pathology PAL Dordrecht Dordrecht The Netherlands; ^18^ Department of Pathology Maastricht University Medical Center Maastricht The Netherlands; ^19^ Department of Pathology Amsterdam University Medical Center Amsterdam The Netherlands; ^20^ Department of Pathology, Cancer Center Amsterdam Vrije Universiteit Amsterdam Amsterdam The Netherlands; ^21^ Department of Cellular Pathology University College London Hospitals NHS Foundation Trust London UK; ^22^ Departments of Pathology and Dermatology Duke University Medical Center Durham North Carolina USA; ^23^ Montevideo Uruguay; ^24^ Department of Pathology Cliniques Universitaires Saint‐Luc Bruxelles Brussels Belgium; ^25^ Department of Gynaecopathology and Breast Pathology Erasme University Hospital‐ULB Brussels Belgium; ^26^ Department of Cytology and Gynecological Pathology Postgraduate Institute of Medical Education and Research Chandigarh India; ^27^ Ball Dermpath Greensboro North Carolina USA; ^28^ Department of Pathology Pathan BV Rotterdam The Netherlands

**Keywords:** carcinoma‐in‐situ, histology, immunohistochemistry, lower genital tract, observer variation, tumour suppressor protein p53, vulvar neoplasm

## Abstract

**Aims:**

Differentiated or HPV‐independent vulvar intraepithelial neoplasia (dVIN) can progress rapidly to invasive cancer and accurate pathological diagnosis is essential to facilitate appropriate interventions. Histological similarities of dVIN with non‐neoplastic lesions, however, often make the diagnosis less reproducible. We investigated among a diverse group of pathologists whether the diagnostic agreement improves with the use of p53 immunohistochemistry (IHC) interpreted using the pattern‐based schema.

**Methods and results:**

Fifty haematoxylin–eosin (HE) stained archival slides (30 dVIN and 20 non‐dysplastic vulvar lesions) were selected and p53‐IHC was performed. Twenty‐four board‐certified pathologists from eight countries first assessed the HE slides alone, and after a washout period, re‐evaluated them alongside the p53‐IHC slides. During both rounds, slides were diagnosed as dVIN, favour dVIN, favour no‐VIN or no‐VIN. p53‐IHC was scored as wild‐type or mutant (diffuse, basal, cytoplasmic or null). Kappa (*κ*) statistics and McNemar's test were used for statistical analyses. Overall diagnostic agreement for dVIN saw a significant increase in the Kappa value (*κ* = 0.6 vs. *κ* = 0.4, *P* = 0.002) when HE and p53‐IHC slides were assessed together compared with histology assessment alone, although the level of agreement remained moderate. For p53‐IHC assessment, overall agreement was substantial (*κ* = 0.7). Diagnoses changing from no‐VIN/favour no‐VIN to dVIN correlated significantly with the identification of a p53‐mutant pattern (*P* < 0.001).

**Conclusions:**

Our findings indicate that p53‐IHC is a robust ancillary tool that can be reproducibly interpreted by pathologists with varying experience levels and supports the routine use of p53‐IHC in cases where dVIN is considered in the differential diagnosis.

## Graphical abstract

Differentiated vulvar intraepithelial neoplasia (dVIN) is a precancerous lesion that can resemble non‐dysplastic lesions histologically. In this study involving 24 pathologists from 8 countries, pattern‐based interpretation of p53 immunohistochemistry improved interobserver agreement for dVIN and reduced rates of ‘indefinite‐for‐dysplasia’ diagnoses compared to histology assessment alone.

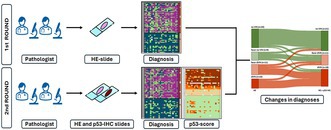

**Abbreviations**
DEVIL: differentiated exophytic vulvar intraepithelial lesion
dVIN: differentiated vulvar intraepithelial neoplasia
HE: hematoxylin and eosin
IHC: immunohistochemistry
VAAD: vulvar acanthosis with altered differentiation
VAM: vulvar aberrant maturation
vaVIN: verruciform acanthotic vulvar intraepithelial neoplasia
VIN: vulvar intraepithelial neoplasia

## Introduction

Vulvar squamous cell carcinoma (VSCC) is one of the rarer gynecologic malignancies; however, its incidence has been rising.^1^ For VSCCs that develop in a field of chronic dermatoses and are unrelated to a human papillomavirus (HPV) infection, two major categories of precursor lesions are recognized^2^, ^3^, ^4^: differentiated vulvar intraepithelial neoplasia (dVIN) or HPV‐independent VIN, and lesions that fall on the spectrum of vulvar acanthosis with abnormal differentiation (VAAD)/differentiated exophytic vulvar intraepithelial lesion (de‐VIL)/vulvar abnormal maturation (VAM)/verruciform acanthotic VIN (vaVIN).^2^, ^3^, ^5^, ^6^, ^7^ While the malignant potential of the latter category of lesions remains unclear, dVIN is well established as an aggressive precursor lesion that may progress to VSCC within 2 years.^8^, ^9^, ^10^ To mitigate the risk of malignant transformation, standard treatment of dVIN is complete surgical excision, and accurate diagnosis is essential for ensuring adequate treatment.^11^, ^12^

The definitive diagnosis of dVIN is established on pathological examination,^4^, ^11^ but histological features of dVIN often overlap with those of non‐neoplastic dermatoses, making diagnosis difficult and inter‐observer agreement suboptimal.^13^, ^14^, ^15^, ^16^, ^17^, ^18^, ^19^ To help distinguish dVIN from its non‐neoplastic mimics, p53 immunohistochemistry is commonly used.^4^, ^12^, ^19^, ^20^ Most HPV‐independent VINs harbour *TP53* mutations, and aberrant p53 expression has been found to correlate strongly with the presence of *TP53* mutation.^21^, ^22^, ^23^ In recent years, a pattern‐based schema for interpreting p53‐IHC has been proposed, which includes four aberrant/mutant patterns (diffuse, basal, cytoplasmic and null‐pattern) and two wild‐type patterns of p53 expression.^24^, ^25^, ^26^, ^27^ Mutant patterns have shown strong concordance with the presence of pathogenic *TP53* mutations, and the interpretation has been reported to be substantially reproducible among experienced Gynaecological Pathologists.^23^, ^24^, ^25^, ^26^, ^28^ While recent studies have assessed agreement in p53‐IHC interpretation among pathologists from different practice settings,^29^, ^30^ no data exist on how p53‐IHC interpretation influences histologic diagnoses of dVIN or resolves ambiguous cases.

We conducted this study with an internationally diverse group of pathologists having varying levels of experience in Gynecologic Pathology with the aim of evaluating (i) the inter‐observer agreement for the interpretation of p53‐IHC following the pattern‐based scoring system, and (ii) the role of p53‐IHC in improving the diagnostic agreement for dVIN and reducing the rates of indefinite diagnoses.

## Materials and Methods

This study was approved by the Institutional Review Board of Erasmus MC and was performed following the guidelines of the Dutch Federation of Biomedical Scientific Societies (www.federa.org/codes‐conduct).

### Preparation of Slide Set for Assessment

Two investigators (SD and PCE‐G) selected a set of 50 haematoxylin–eosin (HE) stained glass slides from a retrospective cohort of vulvar pathology cases (2011–2020) identified from the electronic records of the Department of Pathology, Erasmus MC. For diagnosing dVIN, features of nuclear atypia, such as angulated nuclei, macronucleoli or atypical mitoses were considered essential, and features of abnormal keratinization and architecture were considered supportive.^13^, ^16^, ^31^, ^32^

The selection comprised slides from—(i) vulvar excisions (*n* = 44) and biopsies (*n* = 6), and (ii) standalone lesions (*n* = 27) and lesions adjacent to VSCC (*n* = 23). Thirty of the selected slides had been judged as dVIN and 20 as no‐VIN by the investigators. Of the 30 lesions judged as dVIN, 17 were present adjacent to VSCCs, and for 10 of these, the presence of dVIN was mentioned in the original report. Slides selected from these cases did not have invasive cancer in the same section, to minimise diagnostic bias among participants. All the 13 standalone lesions judged as dVIN by the investigators had been originally diagnosed as dVIN. The selection was enriched for dVIN in keeping with the focus of the study. Both categories of diagnoses, dVIN and no‐VIN, were represented in biopsy and excision specimens (Table S1) to avoid potential bias stemming from associating excision specimens with neoplastic diagnoses. To ensure a comprehensive selection, the investigators included cases bearing prototypical features of dVIN or no‐VIN, as well as those that they considered to be ambiguous for the presence of dysplastic features. The cases that were judged as no‐VIN included lichen sclerosus (*n* = 8), non‐specific reactive/inflammatory changes (*n* = 9), and normal vulvar skin (*n* = 3). All patient data were anonymised, and slides were de‐identified by covering original labels with opaque stickers bearing a new number.

For each slide, p16 and p53 IHC were performed (Supplement 1) at the Pathology laboratory of Erasmus MC. p16‐IHC was scored following the LAST criteria.^33^ None of the cases showed block‐type p16 staining, which ruled out the presence of any HPV‐associated high‐grade squamous intraepithelial lesion (usual VIN) in the selection. p53‐IHC was scored following the pattern‐based schema reported by Kortekaas *et al*.^24^, ^25^

Whole‐slide images (WSIs; .ndpi files) of all HE and IHC slides were acquired using the Nanozoomer (Hamamatsu) scanner under 40× magnification. The areas of the WSIs that were to be assessed by the participants were annotated digitally using the NDPviewer software.

### Participants and Instructions for Assessment

To recruit participants, invitation emails were sent out to members of the (i) International Society for the Study of Vulvovaginal Diseases (ISSVD), (ii) Gynaecological Pathology working group of Rotterdam and (iii) Dutch Working Group for Gynaecological Pathology. In addition, some recognized experts in the field of Gynecologic Pathology from various countries were also invited. To those who expressed interest, first, WSIs of the HE slides were securely transferred along with instructions and forms for assessment (Supplement 2), and participants were asked to diagnose each slide as dVIN, favour dVIN, favour no‐VIN or no‐VIN. After a wash‐out period of around 8 weeks calculated from the point of receiving the results from the first round, WSIs of the p53‐IHC slides, and instructions and forms for the second round of assessment (Supplement 2) were similarly transferred. For this round, participants were asked to assess the HE and p53 slides together, score the p53‐IHC pattern and again provide a diagnosis using the same categories as before. Demographic and practice‐setting related information was gathered from the participants (Table 1). No clinical information nor original diagnoses were provided. To ensure that participants judged slides using their own knowledge/experience, consensus meetings to determine diagnostic criteria were not conducted prior to or between rounds of assessment. Participants did not have access to each other's assessments.

**Table 1:** Participant information

		*n* (%)
Country of practice	Australia	3 (12.6)
	India	1 (4.2)
	Belgium	5 (20.8)
	France	2 (8.3)
	Netherlands	5 (20.8)
	United Kingdom	2 (8.3)
	United States	5 (20.8)
	Uruguay	1 (4.2)
Nature of practice	Academic	15 (62.5)
	Private	5 (20.8)
	Community	4 (16.7)
Years of experience	Median = 15.5 years; Range: 2–36 years	
Subspeciality training in Gyn Pathology	Yes	15 (62.5)
	No	6 (25)
	No information	3 (12.5)

### Statistical Analysis

Data were analysed using R Core Team (2024; Version 4.4.1). Diagnoses and p53‐IHC scores were assessed categorically. Percentages of agreement were computed to obtain absolute measures of agreement. Fleiss' kappa (*κ*) was computed to measure the overall agreement. Cohen's *κ* was computed to measure the agreement between each participant pair, which resulted in 276 *κ*‐values for the diagnoses and p53 scores. *κ*‐values were interpreted as follows: <0.20 = slight, 0.21–0.40 = fair, 0.41–0.60 = moderate, 0.61–0.80 = substantial, or 0.81–1.00 = near‐perfect agreement. Rates of change in diagnoses between the two rounds of assessment were compared using McNemar's test. Chi (*χ* ^2^)‐squared test was used for measuring statistical associations and Spearman's test (*ρ*) for measuring correlation. Bootstrapping (1000 runs) was performed to calculate the 95% confidence intervals (CI). Statistical significance was inferred at a two‐sided *P*‐value < 0.05.

## Results

### Clinical Information

For the cases judged by the investigators as dVIN, the median age was 72.5 years (range: 36–87 years), whereas for those judged as no‐VIN, the median age was 69 years (range: 18–93 years).

### Diagnostic Agreement for dVIN: HE‐Slide Assessment

**Figure 1:**
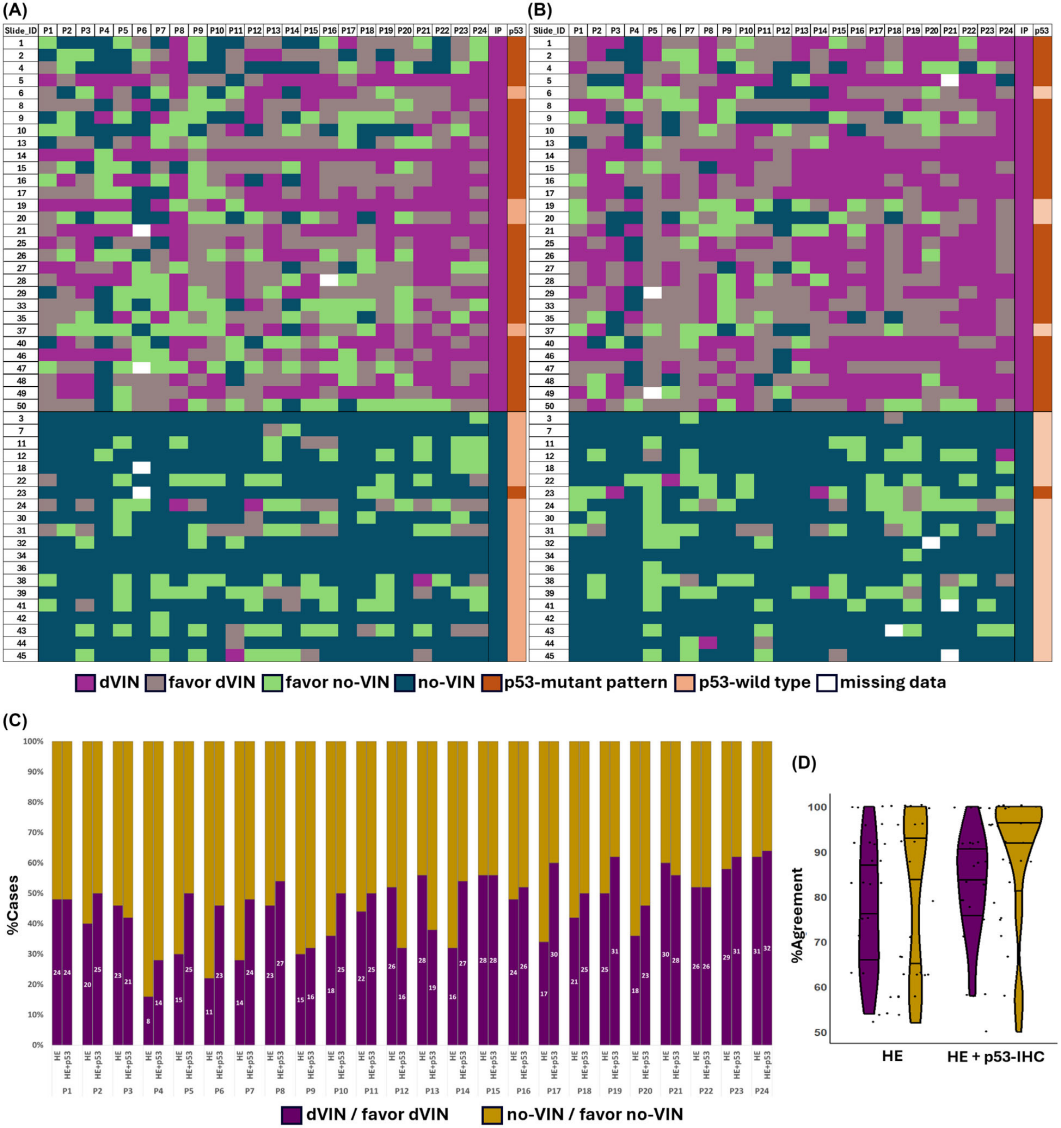
Inter‐observer agreement for the diagnosis of dVIN for both rounds of assessment. Heatmaps represent the diagnoses rendered for each case (rows) per participant (columns), during the **(A)** first round (HE‐slide only) and **(B)** second round (HE and p53‐IHC slides) of assessment. The final column in the heatmap represents p53‐IHC interpretation for each case by the investigating pathologists. P1 through P24 represent the participating pathologists, and IP represents the investigating pathologists. **(C)** Bar charts depict the percentages of the cases (*n* = 50) that were judged as dVIN/favor dVIN or no‐VIN/favor no‐VIN by each participating pathologist during each round of assessment, and **(D)** violin plots represent median, 25th and 75th percentiles of the percentages of agreement for the diagnosis of dVIN/favor dVIN and no‐VIN/favor no‐VIN, during the first (HE) and second (HE + p53‐IHC) rounds of assessment.

Overall agreement for the diagnosis of dVIN, based on the assessment of HE slides, was moderate (*κ* = 0.4; 95% CI = 0.37–0.41), while pairwise agreements ranged from slight to substantial (*κ* = 0.09–0.79). Twenty‐one cases were judged by the majority as dVIN/favour dVIN, with a mean percentage of agreement of 76% (range: 54–100%). A higher percentage of agreement for the diagnosis of dVIN/favour dVIN was observed in cases that can be considered prototypical of dVIN, that is, bearing features of overt nuclear and architectural atypia often discernable under low magnification. Twenty‐eight slides were judged by the majority as no‐VIN/favour no‐VIN, with a mean percentage of agreement of 81% (range: 50%–100%). For one case, there was no majority diagnosis. Variability of the diagnoses rendered per case is presented in Figure 1 and Table 2.

**Table 2:** Majority interpretation of histology and p53 IHC slides

Slide#	Percentages of agreement		
	Histology diagnoses (%)	p53 pattern (%)	Histology and IHC
1	no‐VIN/favour no‐VIN (63)	p53_mut (100)	dVIN/favour dVIN (83)
2	no consensus (50)	p53_mut (100)	dVIN/favour dVIN (79)
3	no‐VIN/favour no‐VIN (100)	p53_wt (92)	no‐VIN/favour no‐VIN (96)
4	no‐VIN/favour no‐VIN (63)	p53_mut (71)	no‐VIN/favour no‐VIN (50)
5	dVIN/favour dVIN (96)	p53_mut (75)	dVIN/favour dVIN (78)
6	dVIN/favour dVIN (63)	p53_wt (79)	dVIN/favour dVIN (58)
7	no‐VIN/favour no‐VIN (96)	p53_wt (100)	no‐VIN/favour no‐VIN (100)
8	dVIN/favour dVIN (79)	p53_mut (96)	dVIN/favour dVIN (83)
9	no‐VIN/favour no‐VIN (58)	p53_wt (67)	no‐VIN/favour no‐VIN (58)
10	no‐VIN/favour no‐VIN (71)	p53_mut (92)	dVIN/favour dVIN (75)
11	no‐VIN/favour no‐VIN (92)	p53_wt (96)	no‐VIN/favour no‐VIN (100)
12	no‐VIN/favour no‐VIN (100)	p53_wt (96)	no‐VIN/favour no‐VIN (92)
13	dVIN/favour dVIN (67)	p53_mut (92)	dVIN/favour dVIN (83)
14	dVIN/favour dVIN (96)	p53_mut (96)	dVIN/favour dVIN (100)
15	dVIN/favour dVIN (63)	p53_mut (100)	dVIN/favour dVIN (88)
16	dVIN/favour dVIN (75)	p53_mut (100)	dVIN/favour dVIN (96)
17	dVIN/favour dVIN (75)	p53_mut (96)	dVIN/favour dVIN (96)
18	no‐VIN/favour no‐VIN (100)	p53_wt (96)	no‐VIN/favour no‐VIN (100)
19	dVIN/favour dVIN (88)	p53_wt (71)	dVIN/favour dVIN (67)
20	no‐VIN/favour no‐VIN (58)	p53_wt (75)	no‐VIN/favour no‐VIN (58)
21	dVIN/favour dVIN (100)	p53_wt (58)	dVIN/favour dVIN (71)
22	no‐VIN/favour no‐VIN (92)	p53_wt (79)	no‐VIN/favour no‐VIN (96)
23	no‐VIN/favour no‐VIN (100)	p53_mut (88)	no‐VIN/favour no‐VIN (88)
24	no‐VIN/favour no‐VIN (54)	p53_wt (100)	no‐VIN/favour no‐VIN (79)
25	dVIN/favour dVIN (92)	p53_mut (100)	dVIN/favour dVIN (92)
26	dVIN/favour dVIN (63)	p53_mut (100)	dVIN/favour dVIN (92)
27	dVIN/favour dVIN (71)	p53_mut (83)	dVIN/favour dVIN (88)
28	dVIN/favour dVIN (83)	p53_mut (92)	dVIN/favour dVIN (88)
29	dVIN/favour dVIN (67)	p53_mut (100)	dVIN/favour dVIN (91)
30	no‐VIN/favour no‐VIN (96)	p53_wt (92)	no‐VIN/favour no‐VIN (100)
31	no‐VIN/favour no‐VIN (63)	p53_wt (71)	no‐VIN/favour no‐VIN (75)
32	no‐VIN/favour no‐VIN (100)	p53_wt (100)	no‐VIN/favour no‐VIN (100)
33	no‐VIN/favour no‐VIN (58)	p53_mut (100)	dVIN/favour dVIN (92)
34	no‐VIN/favour no‐VIN (100)	p53_wt (100)	no‐VIN/favour no‐VIN (100)
35	dVIN/favour dVIN (54)	p53_mut (88)	dVIN/favour dVIN (75)
36	no‐VIN/favour no‐VIN (100)	p53_wt (92)	no‐VIN/favour no‐VIN (100)
37	dVIN/favour dVIN (54)	p53_mut (54)	dVIN/favour dVIN (58)
38	no‐VIN/favour no‐VIN (92)	p53_wt (75)	no‐VIN/favour no‐VIN (88)
39	no‐VIN/favour no‐VIN (88)	p53_wt (71)	no‐VIN/favour no‐VIN (92)
40	dVIN/favour dVIN (63)	p53_mut (71)	dVIN/favour dVIN (79)
41	no‐VIN/favour no‐VIN (92)	p53_wt (100)	no‐VIN/favour no‐VIN (100)
42	no‐VIN/favour no‐VIN (100)	p53_wt (100)	no‐VIN/favour no‐VIN (100)
43	no‐VIN/favour no‐VIN (83)	p53_wt (100)	no‐VIN/favour no‐VIN (100)
44	no‐VIN/favour no‐VIN (96)	p53_wt (92)	no‐VIN/favour no‐VIN (92)
45	no‐VIN/favour no‐VIN (88)	p53_wt (100)	no‐VIN/favour no‐VIN (96)
46	dVIN/favour dVIN (83)	p53_mut (100)	dVIN/favour dVIN (96)
47	no‐VIN/favour no‐VIN (52)	p53_mut (92)	dVIN/favour dVIN (88)
48	dVIN/favour dVIN (83)	p53_mut (100)	dVIN/favour dVIN (79)
49	dVIN/favour dVIN (92)	p53_mut (96)	dVIN/favour dVIN (87)
50	no‐VIN/favour no‐VIN (58)	p53_mut (88)	dVIN/favour dVIN (71)

### Agreement in the Interpretation of p53‐IHC

**Figure 2:**
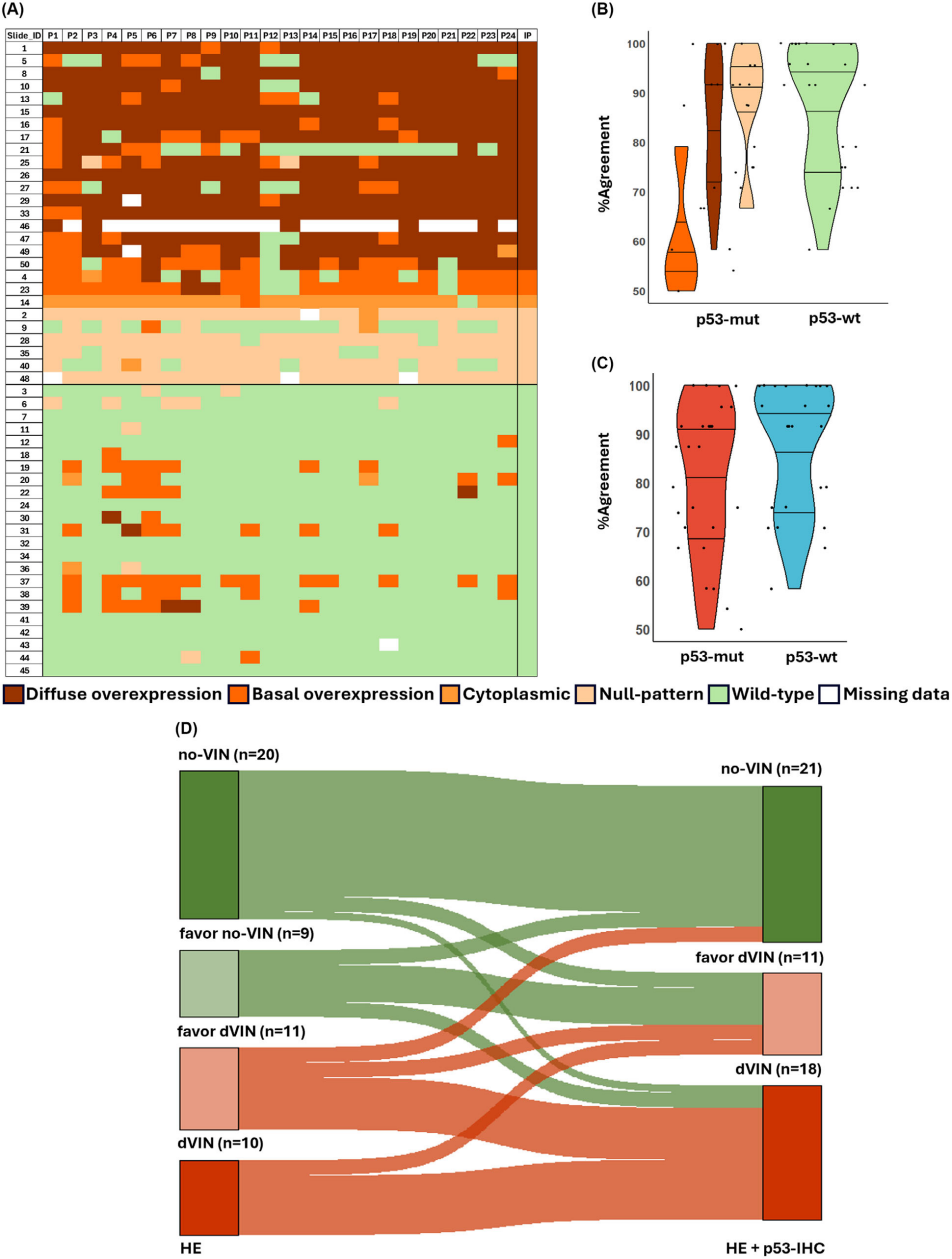
**(A)** Heatmap represents the p53‐patterns for case (rows) judged by each participant columns. The final column in the heatmap represents p53‐IHC interpretation for each case by the investigating pathologists. P1 through P24 represent the participating pathologists and IP represent the investigating pathologists. **(B)** Violin plots represent the median, 25th and 75th percentiles of the percentages of agreement for interpreting the different patterns of p53‐expression, when computed separately and **(C)** when binarized into mutant and wild‐type patterns. **(D)** Sankey plot depicting the changes in majority diagnoses between first (left) and second (right) rounds of assessment.

Overall agreement for the interpretation of p53‐IHC, when the different mutant patterns were analysed separately, was moderate (*κ* = 0.6; 95% CI = 0.58–0.6), with pair‐wise agreements ranging between fair and near‐perfect (*κ* = 0.3–0.9). However, the agreement was substantial when the interpretation categories were binarized as mutant or wild‐type (*κ* = 0.7; 95% CI = 0.65–0.68), with pair‐wise agreements ranging between fair and near‐perfect (*κ* = 0.3–0.9). Twenty‐six slides were interpreted by the majority as one of the mutant patterns of p53‐IHC with a mean percentage of agreement of 91% (range: 54–100%), while 24 slides were interpreted by the majority as wild‐type staining with a mean percentage of agreement of 88% (range: 58%–100%) Variability in the interpretation of p53‐IHC is presented in Figure 2.

Among the patterns of p53‐expression, the median percentage of agreement was highest for wild‐type (92%), followed by null and cytoplasmic (91%) and diffuse overexpression (87%) (Tables S2 and S3). The lowest median percentage of agreement was observed for basal overexpression (56%), which seemed to be often confounded with a wild‐type pattern (Tables S4–S7).

### Diagnostic Agreement for dVIN: HE and p53‐IHC Assessment

**Figure 3:**
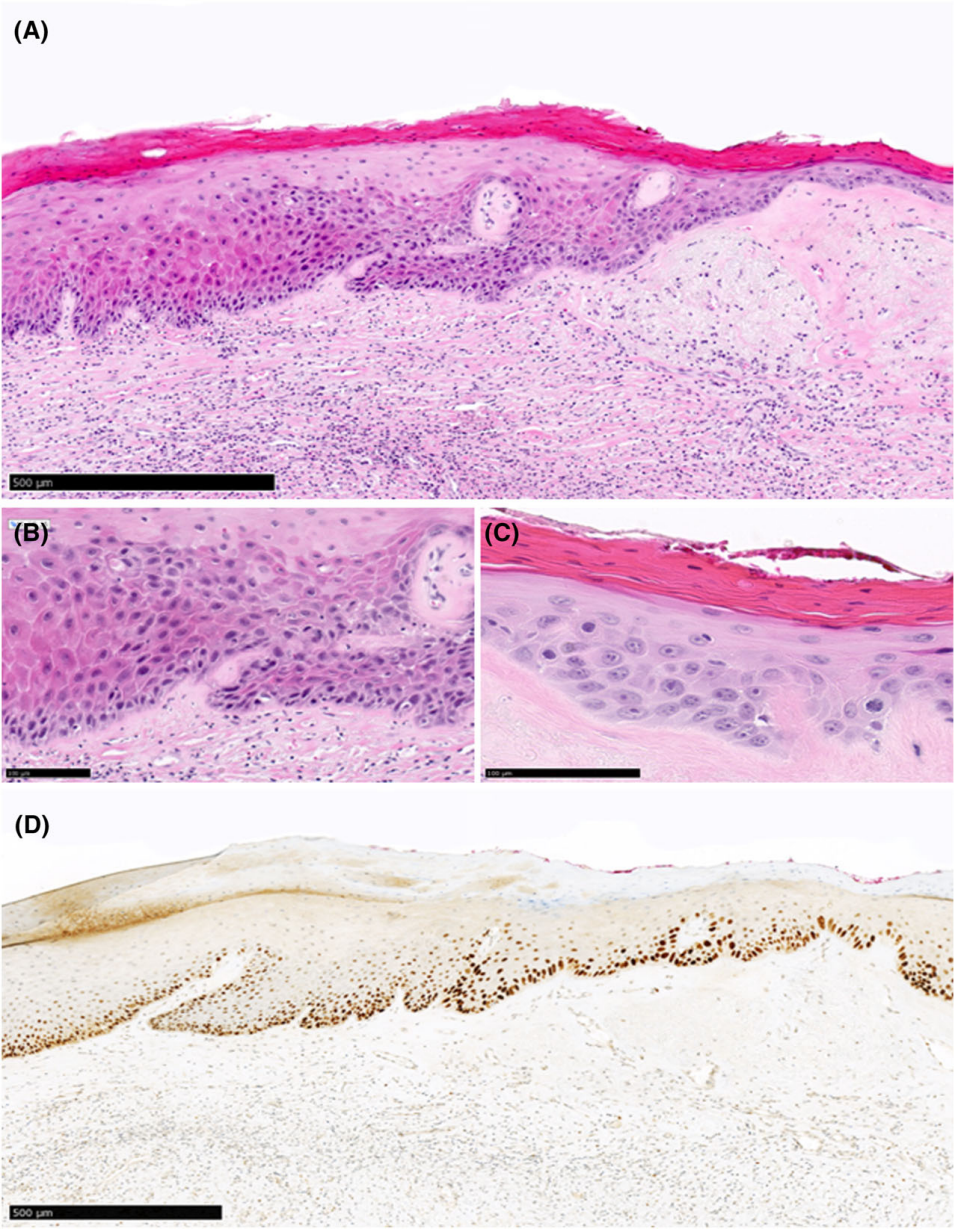
Example of a case (Slide_25) judged as dVIN with >90% agreement during both rounds of assessment. **(A)** Histologic features associated with dVIN (thickened epithelium, eosinophilic appearance, hyper and parakeratosis) can be observed under low magnification. Examination under higher magnification **(B, C)** shows heterogeneity in nuclear size and shape, mitotic figure, individual cell keratinization and cobblestone appearance **(D)** p53‐IHC was judged as diffuse overexpression with >90% agreement.

**Figure 4:**
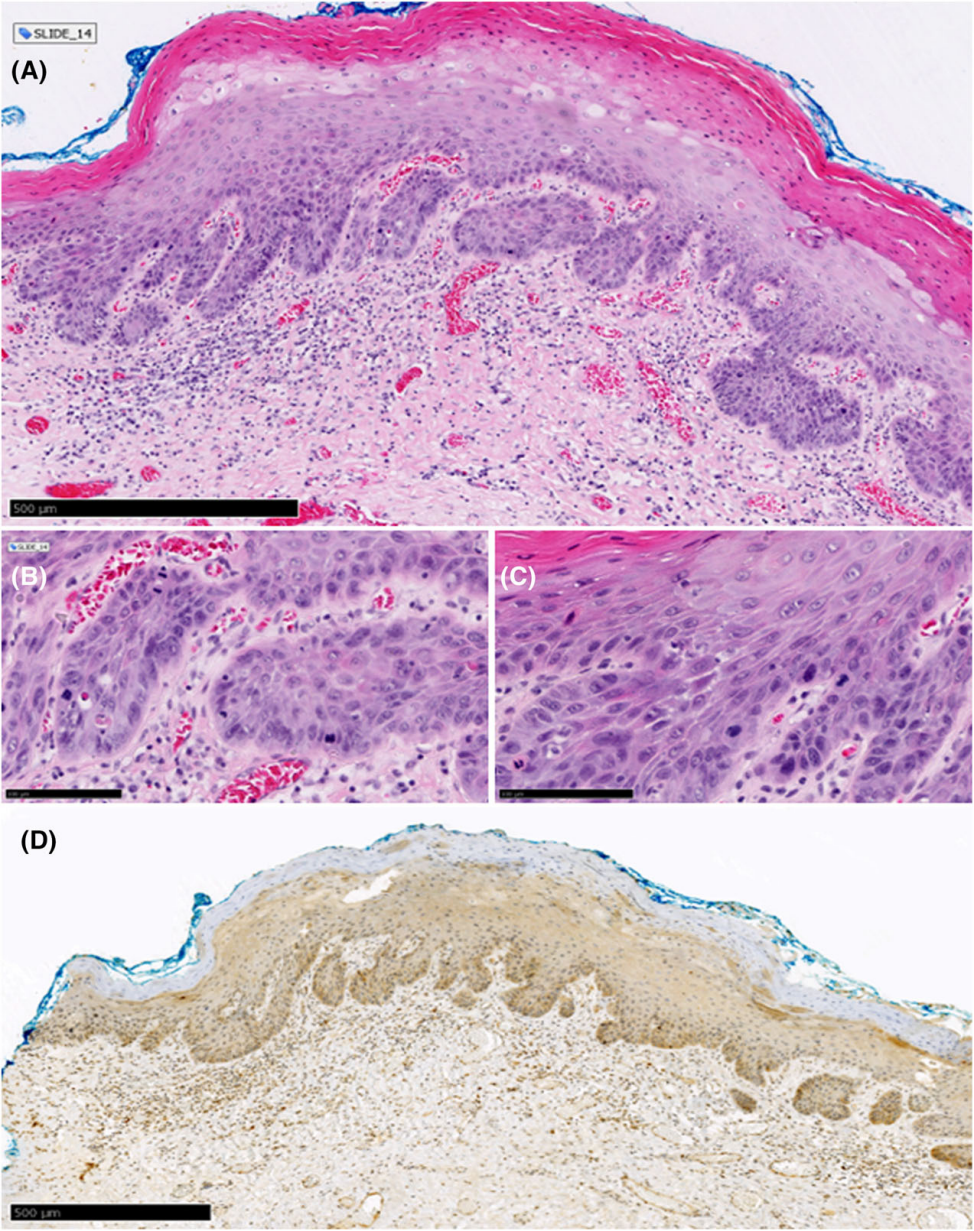
Example of a case (Slide_14) judged as dVIN with >90% agreement during both rounds of agreement. **(A)** Histologic features associated with dVIN (thickened epithelium, eosinophilic appearance, hyper and parakeratosis) can be observed under low magnification. Examination under higher magnification **(B, C)** shows extensive nuclear atypia, hyperchromatic nuclei and individual cell keratinization **(D)** p53‐IHC was judged as cytoplasmic expression with >90% agreement.

Overall agreement for the diagnosis of dVIN during the second round of assessment was moderate (*κ* = 0.6; 95% CI = 0.55–0.58), while pairwise agreements ranged from slight to substantial (*κ* = 0.09–0.79). The change in the level of overall and pairwise agreement from the histology‐only diagnoses was statistically significant (*P* < 0.01). Twenty‐seven slides were judged by the majority as dVIN/favour dVIN, with a mean percentage of agreement of 82% (range: 58%–100%). Twenty‐three slides were judged by the majority as no‐VIN/favour no‐VIN, with a mean percentage of agreement of 89% (range: 50%–100%) (Table 2). Figures 3 and 4 illustrate examples of cases judged as dVIN and showing a mutant pattern of p53 expression (diffuse overexpression and cytoplasmic respectively) with >90% agreement.

### Role of p53‐IHC Assessment in Making a Diagnosis

**Figure 5:**
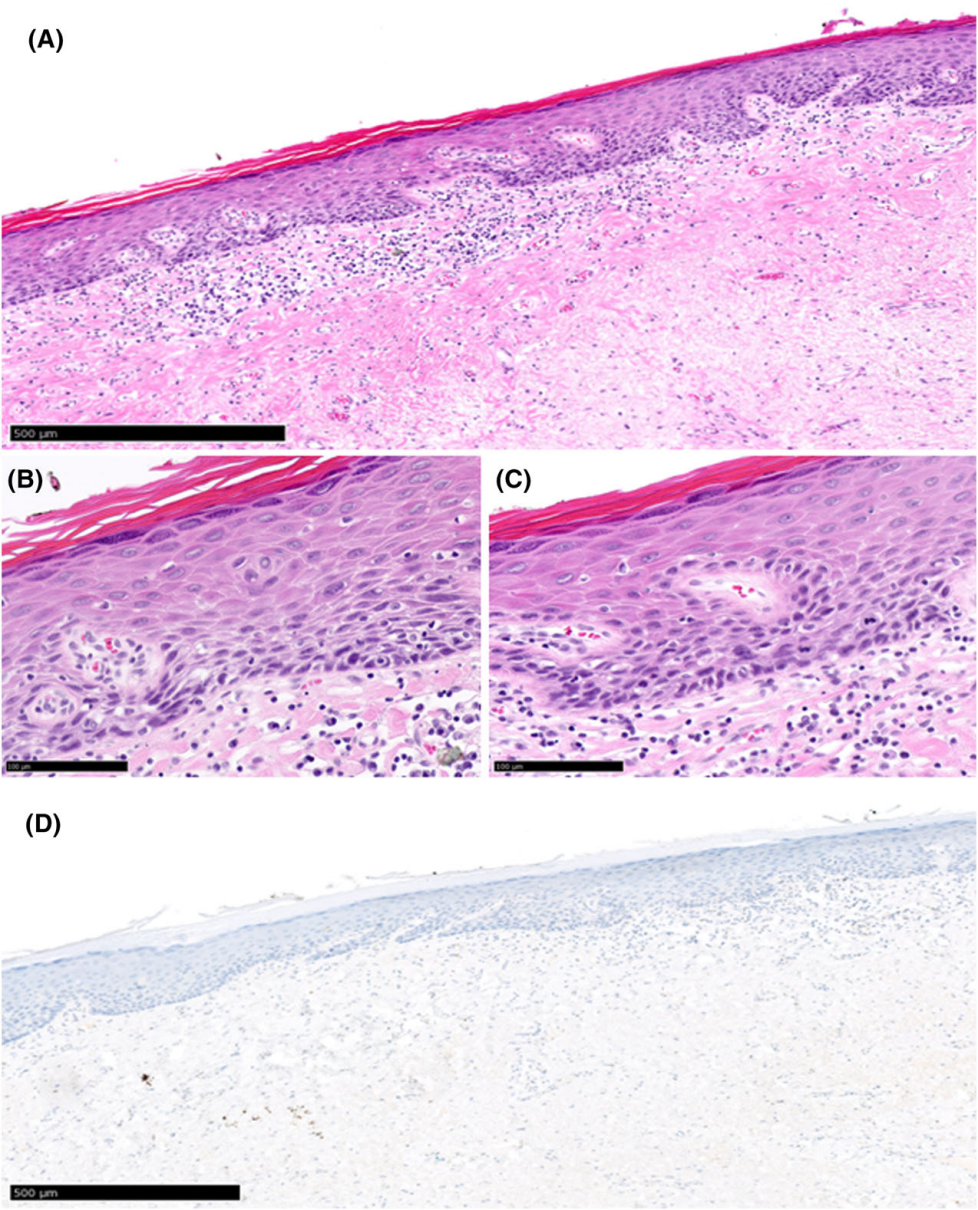
Case (Slide_2) that did not have a majority diagnosis during the first round of assessment (50% diagnosed as dVIN or favor dVIN and 50% diagnosed as no‐VIN or favor no‐VIN). **(A)** Histological features of atypia are barely observable under low magnification. **(B, C)** Higher magnification examination reveals subtle nuclear atypia, that is, nuclear angulation and hyperchromatic nuclei **(D)** p53‐IHC was judged as null‐pattern with 100% agreement, and the majority diagnosis changed to dVIN/favor dVIN during the second round of assessment; inset shows an area with stromal p53‐expression.

**Figure 6:**
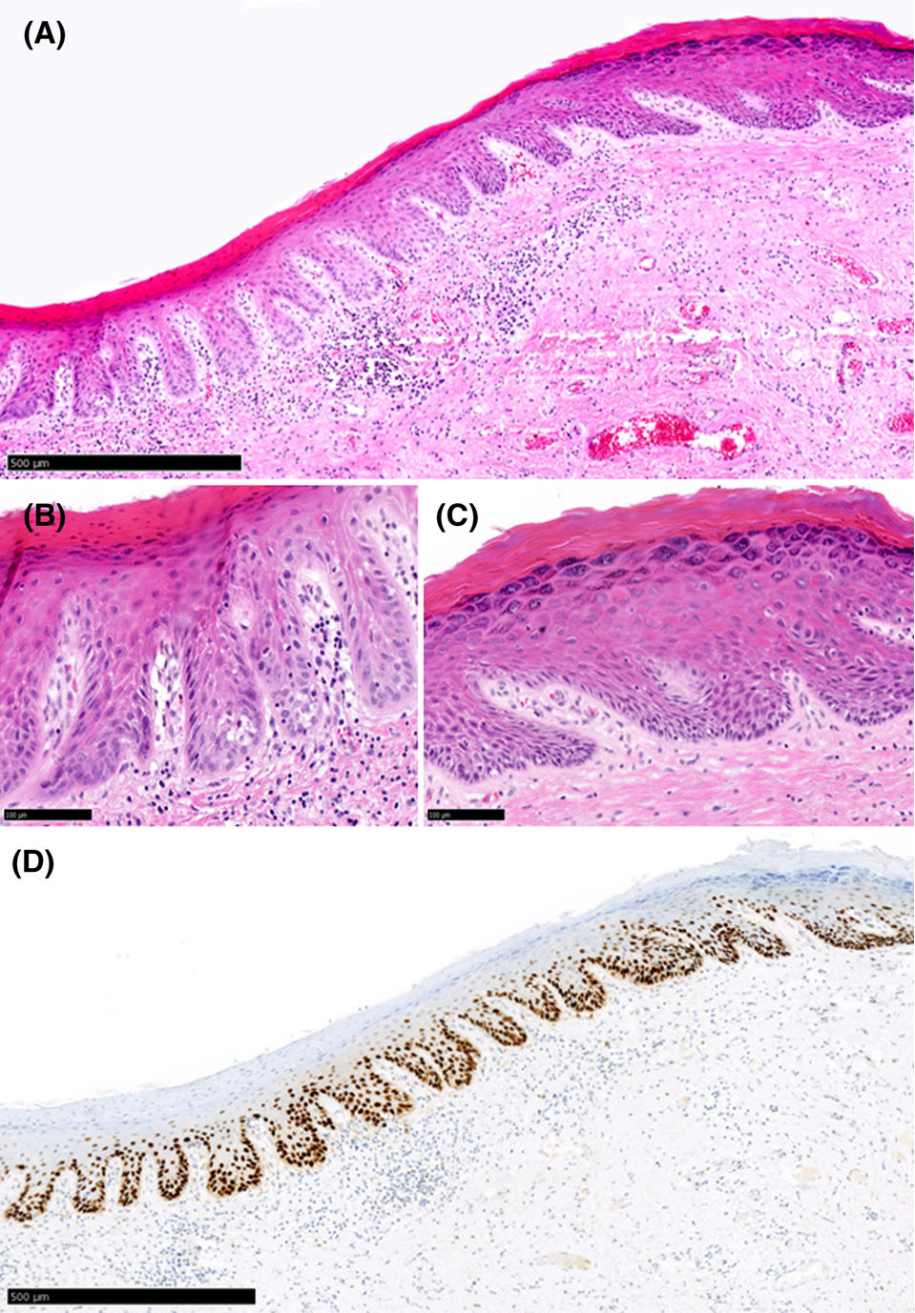
Example of a case (Slide_1) where the majority diagnosis changed from no‐VIN/favor no‐VIN to dVIN/favor dVIN during the second round of assessment **(A)** Low power examination shows prominent parakeratosis, eosinophilic appearance of the epithelial cells, and occasional hyperchromatic nuclei **(B, C)** Higher magnification examination reveals features of nuclear atypia and individual cell keratinization **(D)** p53‐IHC was judged as diffuse overexpression >90% agreement.

Cases that lacked overt features of histological atypia were often categorized as no‐VIN/favour no‐VIN during the first round of assessment, with the diagnosis changing to dVIN/favour dVIN upon the assessment of a mutant pattern of p53‐IHC during the second round. In these cases, features of atypia could be detected only upon careful examination under high magnification. Examples of such cases are illustrated in Figures 5 and 6, where examination under higher magnification reveals the presence of occasional hyperchromatic or enlarged nuclei, macronucleoli, mitotic figures, parakeratosis and ‘cobblestone‐appearance’ caused by a combination of abnormal keratinization and spongiosis.

Majority diagnoses changed significantly during the second round (22 [44%] cases; *P* = 0.03). This included changes from favour no‐VIN/no‐VIN to dVIN/favour dVIN in 8 cases and from favour dVIN to no‐VIN in 2 cases (Figure 2). Overall, an increase in the diagnoses of dVIN (5.7%) and proportion of definitive diagnoses that is dVIN/no‐VIN (4.7%) was observed. A change in the diagnosis from no‐VIN/favour no‐VIN to dVIN/favour dVIN correlated strongly (ρ = 0.89) with the assessment of a mutant pattern of p53‐IHC. Diagnoses rendered in the second round showed strong correlation (median *ρ* = 0.72) with p53‐IHC assessment, that is a diagnosis of dVIN/favour dVIN was associated with the assessment of a mutant pattern of p53‐IHC. The case that had no majority diagnosis during the first round of assessment was judged as dVIN by the majority during the second round, as it showed a null pattern on p53‐IHC (Figure 5). Changing a diagnosis upon IHC assessment did not show any correlation with the years of experience or nature of practice of the pathologist. Changes in diagnostic categories per pathologist and concordance with the p53‐IHC assessment are presented in Table 3.

**Table 3:** Changes in diagnoses and concordance with p53 assessment per participant

Diagnostic category switches				
Pathologists	dVIN/favour dVIN to no‐VIN/favour no‐VIN	Concordant with p53‐IHC (%)	no‐VIN/favour no‐VIN to dVIN/favour dVIN	Concordant with p53‐IHC (%)
1	6	6 (100)	6	6 (100)
2	5	2 (40)	10	10 (100)
3	6	5 (83)	4	4 (100)
4	0	0 (0)	6	6 (100)
5	2	1 (50)	12	10 (83)
6	3	0 (0)	12	12 (100)
7	2	1 (50)	11	11 (100)
8	1	1 (100)	5	5 (100)
9	6	3 (50)	7	6 (86)
10	0	0 (0)	7	7 (100)
11	4	3 (75)	6	6 (100)
12	12	12 (100)	3	3 (100)
13	11	11 (100)	2	2 (100)
14	3	3 (100)	14	14 (100)
15	2	2 (100)	2	2 (100)
16	5	5 (100)	6	6 (100)
17	0	0 (0)	12	11 (92)
18	2	1 (50)	7	6 (86)
19	0	0 (0)	6	4 (67)
20	2	2 (100)	7	7 (100)
21	2	2 (100)	2	2 (100)
22	1	1 (100)	1	1 (100)
23	1	1 (100)	3	3 (100)
24	4	4 (100)	5	5 (100)

## Discussion

Pathologists widely acknowledge the challenges of diagnosing dVIN, and previous studies have attempted to identify histological features that can improve the diagnostic reproducibility and accuracy.^16^, ^18^, ^32^ p53‐IHC is a commonly used tool in clinical practice, and a pattern‐based schema has been proposed for its interpretation, which has been reported to correlate well with the molecular status of the lesions.^25^ As IHC is used as an ancillary tool to confirm or rule out a provisional diagnosis made on histological examination, it is important that the marker can be objectively interpreted.

In this study conducted with an internationally diverse group of pathologists having varying levels of experience in Gynaecological Pathology, we observed that agreement for p53‐IHC interpretation was moderate when the individual patterns were analysed separately and was substantial when the patterns were binarized as mutant and wild‐type. It can be argued that accurate identification of an expression pattern as mutant can be more impactful on clinical decision making than distinguishing between the individual mutant patterns.

Although the use of p53‐IHC led to an increase in the Kappa value of the diagnostic agreement for dVIN, the level of agreement remained moderate. However, reading HE and p53‐IHC slides together reduced the proportion of cases that received an indefinite diagnosis (favour dVIN or favour no‐VIN) during a histology‐only assessment and increased the percentage of dVIN diagnoses. When HE and p53‐IHC slides were assessed together, the general trend was to render a diagnosis that was aligned with the recorded p53‐IHC pattern. We believe, therefore, our findings indicate that p53‐IHC is a valuable tool that can be reproducibly interpreted by pathologists in the context of a dVIN diagnosis.

A change in diagnosis to dVIN was particularly observed for cases (examples illustrated in Figures 5 and 6) that lacked the' prototypical' histology of dVIN, where detecting features of cytological or architectural atypia requires close observation under higher magnification. These cases can be considered more' subtle' examples of dVIN and our findings suggest the use of p53‐IHC can be helpful to prevent a missed diagnosis in such cases.

The question arises as to which cases benefit most from the use of p53‐IHC. In the majority of' prototypical' dVIN cases, that is those with overt features of atypia, p53‐IHC typically exhibits a mutant pattern. In such instances, if dVIN is already strongly suspected based on histological assessment, the diagnosis remains unchanged upon recording a mutant pattern of p53‐IHC. Even if p53‐IHC shows a wild‐type pattern in such a case (a subset of dVIN shows wild‐type p53‐expression^4^, ^34^), it is unlikely the diagnosis will change considering the histology was strongly supportive of dVIN. It may therefore seem that p53‐IHC is most valuable where dVIN is suspected but the diagnosis cannot be definitively established based on histological features alone. However, pathologists do not necessarily always agree on what constitutes a ‘prototypical’ histotype, likely due to differences in training, experience and reliance on different diagnostic criteria.^35^ This variability complicates standardizing which cases require additional IHC. Since missing a diagnosis of dVIN subjects the patient to a risk of developing invasive cancer, we support the routine use of p53‐IHC in all cases of suspected dVIN.

Among the different mutant patterns of p53‐expression, we observed basal overexpression to have the lowest agreement, and participants often recorded this as wild‐type. Similar findings were reported by a recent study investigating interrater agreement for p53‐IHC interpretation in VSCCs, where they observed a scattering of p53 staining in the periphery of the tumour often confounding the distinction between wild‐type and basal overexpression patterns.^29^ Furthermore, another study reported pathologists frequently interpreting a basal overexpression pattern in both dysplastic and non‐dysplastic vulvar lesions, which were wild‐type on *TP53* sequencing.^30^ Although basal overexpression tends to occur in a minority of dVINs,^30^, ^36^ evidently, its distinction from wild‐type pattern needs to be better delineated. More data on *TP53*‐sequencing‐p53‐IHC correlation gathered from larger, multi‐institutional cohorts and educational materials for pathologists with example images can be helpful in this regard. Equally important is the standardization of specimen handling and staining protocols, as a multitude of pre‐analytical factors, for example, fixation times or choice of antibody clones, can significantly impact IHC interpretation and diagnostic reliability. It is also important to recognize the limitations of p53‐IHC as a diagnostic tool. In our study cohort, one case of lichen sclerosus exhibited basal overexpression, and four dVIN cases showed wild‐type p53 staining. In such instances, particularly when dVIN is suspected but p53 appears wild‐type, additional immunohistochemical markers such as CK17,^37^ GATA3^38^ or SOX2^39^ may provide supportive diagnostic information.

There are certain limitations to our study. Our study design may not reflect a typical clinical scenario in which a pathologist makes a diagnosis, because participants made a diagnosis from a predefined set of options on a set of cases selected from a retrospective cohort with no clinical information, with only a single slide per case. We included a diverse group of pathologists in our study; however, all of them sign out vulvar pathology cases regularly in their practice, and it might be of value evaluating the role of p53‐IHC in influencing diagnosis among general pathologists. We did not use a ‘gold‐standard’ diagnosis in this study as our aim was to evaluate the reproducibility of p53‐IHC interpretation among a diverse group of pathologists, and not to compare their scores with a ‘gold‐standard’. Although correlating the IHC patterns with the *TP53* mutation status of the lesions could have been valuable, molecular testing was not performed as this was not the primary focus of the study.

In conclusion, we observed that p53‐IHC is a robust ancillary tool that can be interpreted with substantial agreement by pathologists from diverse practice settings and having varying levels of experience, and adds value as a part of routine diagnostic workup for dVIN. Although p53‐IHC may not alter the diagnosis in more prototypical cases, it plays a crucial role in more subtle cases of dVIN, where histological examination alone might not provide a definitive diagnosis. Our findings support the routine use of p53‐IHC in all cases where dVIN is considered in the differential diagnosis, to enhance diagnostic accuracy and prevent missed diagnoses.

## Author contribution

Conceptualization: S.D., P.C.E‐G, S.K., F.J.v.K. Data curation: All authors. Formal analysis: S.D., P.C.E.G. Investigation: All authors. Visualization: S.D. Writing‐original draft: S S.D., P.C.E‐G, S.K., F.J.v.K. Writing–review and editing: All authors. Supervision: F.J.v.K, P.C.E.G. Methodology: S.D., P.C.E.G, S.K., F.J.v.K. Funding acquisition: F.J.v.K. Project administration: F.J.v.K.

## Funding information

No external funding was received for this study.

## Conflicts of interest

The authors declare no conflicts of interest.

## Supporting information


**Table S1.** Distribution of included cases
**Table S2.** Percentages of agreement for each pattern of p53‐immunohistochemistry
**Table S3.** p53‐immunohistochemistry interpretation rendered per slide
**Table S4.** Most commonly reported patterns for slides interpreted as diffuse overexpression by the majority
**Table S5.** Most commonly reported patterns for slides interpreted as null‐pattern by the majority
**Table S6.** Most commonly reported patterns for slides interpreted as cytoplasmic pattern by the majority
**Table S7.** Most commonly reported patterns for slides interpreted as basal overexpression by the majority

**Data S2.** Immunohistochemistry (IHC) protocol.

**Data S3.** Differentiated vulvar intraepithelial neoplasia (dVIN): inter‐observer variability in the histological diagnosis and interpretation of p53‐immunohistochemistry.

## Data Availability

Anonymized data associated with this manuscript including whole slide images may be shared upon reasonable request following institutional policies. Requests should be directed to the corresponding author and Prof. F.J. van Kemenade (f.vankemenade@erasmusmc.nl).
