# Supplementary material for: Use of p53 immunohistochemistry can improve diagnostic agreement for differentiated vulvar intraepithelial neoplasia (dVIN): an international reproducibility study
Source: Histopathology. 2025 Aug 5;88(2):414–28. doi: 10.1111/his.15524 (PMC12703432; doi:10.1111/his.15524)
Supplement: Supplementary file 2 — Data S2. Immunohistochemistry (IHC) protocol. [file HIS-88-414-s002.pdf]

Immunohistochemistry (IHC) protocol

For IHC, serial sections of 4 μm thickness were prepared from formalin fixed paraffin embedded (FFPE) tissues, and were mounted on adhesive glass slides. Immunohistochemistry was performed using an automated, validated and accredited staining system (Ventana Benchmark ULTRA, Ventana Medical Systems, Tucson, AZ, USA).

De-paraffinization was performed according to the BenchMark Ultra protocol. Antigen retrieval was performed using CC1 antigen retrieval solution (ref. 950-124, Ventana Medical Systems, Inc) for 64 minutes. For detection, UltraView universal DAB (ref. 760-500, Ventana Medical Systems, Inc.) or Ultraview Universal Alkaline Red detection kit (ref. 760-501) were used. Next, the specimens were incubated with the respective primary antibodies. Amplification was performed using Kit ref: 760-080. This was followed by counterstaining with haematoxylin II (ref: 790-2208, Ventana Medical Systems, Inc). Each slide contained an appropriate positive control.

Details of the primary antibodies, detection, and amplification are tabulated below:

Antibodies	Clone	Supplier	Reference number	Detection	Incubation with primary antibody	Amplification
CintecR© p16	--	Ventana	805-2020	UltraView DAB	37°C for 12 min.	Yes
p53	Bp53 -11	Ventana	760-2542	UltraView DAB	37°C for 4 min.	Yes
