# Supplementary material for: Use of p53 immunohistochemistry can improve diagnostic agreement for differentiated vulvar intraepithelial neoplasia (dVIN): an international reproducibility study
Source: Histopathology. 2025 Aug 5;88(2):414–28. doi: 10.1111/his.15524 (PMC12703432; doi:10.1111/his.15524)
Supplement: Supplementary file 3 — Data S3. Differentiated vulvar intraepithelial neoplasia (dVIN): inter‐observer variability in the histological diagnosis and interpretation of p53‐immunohistochemistry. [file HIS-88-414-s003.pdf]

Differentiated vulvar intraepithelial neoplasia (dVIN): Inter-observer variability in the histological diagnosis and interpretation of p53-immunohistochemistry

Instructions: Please assess the areas marked on the slide, or the whole slide if there are no markings, and provide a diagnosis based on the options provided. Additional comments can be added in the boxes below (optional).

SLIDE 1

○ **dVIN** ○ **favor dVIN** ○ **favor no-VIN** ○ **no-VIN**

COMMENTS

SLIDE 2

○ **dVIN** ○ **favor dVIN** ○ **favor no-VIN** ○ **no-VIN**

COMMENTS

SLIDE 3

○ **dVIN** ○ **favor dVIN** ○ **favor no-VIN** ○ **no-VIN**

COMMENTS

SLIDE 4

◯ dVIN ◯ favor dVIN ◯ favor no-VIN ◯ no-VIN

COMMENTS

SLIDE 5

◯ dVIN ◯ favor dVIN ◯ favor no-VIN ◯ no-VIN

COMMENTS

SLIDE 6

◯ dVIN ◯ favor dVIN ◯ favor no-VIN ◯ no-VIN

COMMENTS

SLIDE 7

◯ dVIN ◯ favor dVIN ◯ favor no-VIN ◯ no-VIN

COMMENTS

SLIDE 8

◯ dVIN ◯ favor dVIN ◯ favor no-VIN ◯ no-VIN

COMMENTS

SLIDE 9

◯ dVIN ◯ favor dVIN ◯ favor no-VIN ◯ no-VIN

COMMENTS

SLIDE 10

◯ dVIN ◯ favor dVIN ◯ favor no-VIN ◯ no-VIN

COMMENTS

SLIDE 11

◯ dVIN ◯ favor dVIN ◯ favor no-VIN ◯ no-VIN

COMMENTS

SLIDE 12

○ dVIN ○ favor dVIN ○ favor no-VIN ○ no-VIN

COMMENTS

SLIDE 13

○ dVIN ○ favor dVIN ○ favor no-VIN ○ no-VIN

COMMENTS

SLIDE 14

○ dVIN ○ favor dVIN ○ favor no-VIN ○ no-VIN

COMMENTS

SLIDE 15

○ dVIN ○ favor dVIN ○ favor no-VIN ○ no-VIN

COMMENTS

SLIDE 16

○ dVIN ○ favor dVIN ○ favor no-VIN ○ no-VIN

COMMENTS

SLIDE 17

○ dVIN ○ favor dVIN ○ favor no-VIN ○ no-VIN

COMMENTS

SLIDE 18

○ dVIN ○ favor dVIN ○ favor no-VIN ○ no-VIN

COMMENTS

SLIDE 19

○ dVIN ○ favor dVIN ○ favor no-VIN ○ no-VIN

COMMENTS

SLIDE 20

○ dVIN ○ favor dVIN ○ favor no-VIN ○ no-VIN

COMMENTS

SLIDE 21

○ dVIN ○ favor dVIN ○ favor no-VIN ○ no-VIN

COMMENTS

SLIDE 22

○ dVIN ○ favor dVIN ○ favor no-VIN ○ no-VIN

COMMENTS

SLIDE 23

○ dVIN ○ favor dVIN ○ favor no-VIN ○ no-VIN

COMMENTS

SLIDE 24

○ dVIN ○ favor dVIN ○ favor no-VIN ○ no-VIN

COMMENTS

SLIDE 25

○ dVIN ○ favor dVIN ○ favor no-VIN ○ no-VIN

COMMENTS

SLIDE 26

○ dVIN ○ favor dVIN ○ favor no-VIN ○ no-VIN

COMMENTS

SLIDE 27

○ dVIN ○ favor dVIN ○ favor no-VIN ○ no-VIN

COMMENTS

SLIDE 28

◯ dVIN ◯ favor dVIN ◯ favor no-VIN ◯ no-VIN

COMMENTS

SLIDE 29

◯ dVIN ◯ favor dVIN ◯ favor no-VIN ◯ no-VIN

COMMENTS

SLIDE 30

◯ dVIN ◯ favor dVIN ◯ favor no-VIN ◯ no-VIN

COMMENTS

SLIDE 31

◯ dVIN ◯ favor dVIN ◯ favor no-VIN ◯ no-VIN

COMMENTS

SLIDE 32

◯ dVIN ◯ favor dVIN ◯ favor no-VIN ◯ no-VIN

COMMENTS

SLIDE 33

◯ dVIN ◯ favor dVIN ◯ favor no-VIN ◯ no-VIN

COMMENTS

SLIDE 34

◯ dVIN ◯ favor dVIN ◯ favor no-VIN ◯ no-VIN

COMMENTS

SLIDE 35

◯ dVIN ◯ favor dVIN ◯ favor no-VIN ◯ no-VIN

COMMENTS

SLIDE 36

○ dVIN ○ favor dVIN ○ favor no-VIN ○ no-VIN

COMMENTS

SLIDE 37

○ dVIN ○ favor dVIN ○ favor no-VIN ○ no-VIN

COMMENTS

SLIDE 38

○ dVIN ○ favor dVIN ○ favor no-VIN ○ no-VIN

COMMENTS

SLIDE 39

○ dVIN ○ favor dVIN ○ favor no-VIN ○ no-VIN

COMMENTS

SLIDE 40

○ dVIN ○ favor dVIN ○ favor no-VIN ○ no-VIN

COMMENTS

SLIDE 41

○ dVIN ○ favor dVIN ○ favor no-VIN ○ no-VIN

COMMENTS

SLIDE 42

○ dVIN ○ favor dVIN ○ favor no-VIN ○ no-VIN

COMMENTS

SLIDE 43

○ dVIN ○ favor dVIN ○ favor no-VIN ○ no-VIN

COMMENTS

Erasmus MC
Universitair Medisch Centrum Rotterdam

SLIDE 44

○ dVIN ○ favor dVIN ○ favor no-VIN ○ no-VIN

COMMENTS

SLIDE 45

○ dVIN ○ favor dVIN ○ favor no-VIN ○ no-VIN

COMMENTS

SLIDE 46

○ dVIN ○ favor dVIN ○ favor no-VIN ○ no-VIN

COMMENTS

SLIDE 47

○ dVIN ○ favor dVIN ○ favor no-VIN ○ no-VIN

COMMENTS

SLIDE 48

◯ dVIN ◯ favor dVIN ◯ favor no-VIN ◯ no-VIN

COMMENTS

SLIDE 49

◯ dVIN ◯ favor dVIN ◯ favor no-VIN ◯ no-VIN

COMMENTS

SLIDE 50

◯ dVIN ◯ favor dVIN ◯ favor no-VIN ◯ no-VIN

COMMENTS

--*END OF HISTOLOGICAL ASSESSMENT*--

Erasmus MC
Universitair Medisch Centrum Rotterdam

QUESTIONNAIRE - PARTICIPANT INFORMATION

Kindly fill out the following questionnaire.
Information gathered through this questionnaire will be used solely for the purpose of preparation of the manuscript. Assessments of all participants will be anonymized.

Name of participant

Country(ies) of practice

Length of practicing experience in years (Excluding years in training)

Specific sub-specilisation training / Fellowship in Gynecologcial Pathology ○ **Yes** ○ **No**

Type of practice ○ **Academic** ○ **Community** ○ **Private**

Proportion of Gynecological Pathology cases in practice ○ **< 50%** ○ **> 50%**

Degree(s)

ORCID iD (if any)

Current affiliation(s) and e-mail (for publication)

---------------------------------*OUR SINCERE THANKS FOR YOUR PARTICIPATION*---------------------------------------

Differentiated vulvar intraepithelial neoplasia (dVIN): Inter-observer variability in the histological diagnosis and interpretation of p53-immunohistochemistry

Instructions

There are two steps for the assessment.

1. Please assess the p53 immunohistochemistry (IHC) slide of each case, and score the staining pattern based on the options provided. For scoring, kindly refer to the p53-IHC scoring guide.
2. Please assess the hematoxylin-eosin stained slide and the p53-IHC slide of each case, and provide a diagnosis based on the options provided.
   Additional comments can be added in the boxes below (optional).

SLIDE 1

p53-IHC PATTERN

◯ **Basal to para-basal/diffuse overexpression**

◯ **Basal overexpression**

◯ **Null-pattern**

◯ **Cytoplasmic**

◯ **Wild-type**

DIAGNOSIS

◯ **dVIN** ◯ **favor dVIN** ◯ **favor no-VIN** ◯ **no-VIN**

COMMENTS

SLIDE 2

p53-IHC PATTERN

◯ **Basal to para-basal/diffuse overexpression**

◯ **Basal overexpression**

◯ **Null-pattern**

◯ **Cytoplasmic**

◯ **Wild-type**

DIAGNOSIS

- ○ dVIN
- ○ favor dVIN
- ○ favor no-VIN
- ○ no-VIN

COMMENTS

SLIDE 3

p53-IHC PATTERN

- ○ Basal to para-basal/diffuse overexpression
- ○ Basal overexpression
- ○ Null-pattern
- ○ Cytoplasmic
- ○ Wild-type

DIAGNOSIS

- ○ dVIN
- ○ favor dVIN
- ○ favor no-VIN
- ○ no-VIN

COMMENTS

SLIDE 4

p53-IHC PATTERN

- ○ Basal to para-basal/diffuse overexpression
- ○ Basal overexpression
- ○ Null-pattern
- ○ Cytoplasmic
- ○ Wild-type

DIAGNOSIS

- ◯ dVIN
- ◯ favor dVIN
- ◯ favor no-VIN
- ◯ no-VIN

COMMENTS

SLIDE 5

p53-IHC PATTERN

- ◯ Basal to para-basal/diffuse overexpression
- ◯ Basal overexpression
- ◯ Null-pattern
- ◯ Cytoplasmic
- ◯ Wild-type

DIAGNOSIS

- ◯ dVIN
- ◯ favor dVIN
- ◯ favor no-VIN
- ◯ no-VIN

COMMENTS

SLIDE 6

p53-IHC PATTERN

- ◯ Basal to para-basal/diffuse overexpression
- ◯ Basal overexpression
- ◯ Null-pattern
- ◯ Cytoplasmic
- ◯ Wild-type

DIAGNOSIS

○ dVIN ○ favor dVIN ○ favor no-VIN ○ no-VIN

COMMENTS

SLIDE 7

p53-IHC PATTERN

○ Basal to para-basal/diffuse overexpression

○ Basal overexpression

○ Null-pattern

○ Cytoplasmic

○ Wild-type

DIAGNOSIS

○ dVIN ○ favor dVIN ○ favor no-VIN ○ no-VIN

COMMENTS

SLIDE 8

p53-IHC PATTERN

○ Basal to para-basal/diffuse overexpression

○ Basal overexpression

○ Null-pattern

○ Cytoplasmic

○ Wild-type

DIAGNOSIS

○ dVIN ○ favor dVIN ○ favor no-VIN ○ no-VIN

COMMENTS

SLIDE 9

p53-IHC PATTERN

○ Basal to para-basal/diffuse overexpression

○ Basal overexpression

○ Null-pattern

○ Cytoplasmic

○ Wild-type

DIAGNOSIS

○ dVIN ○ favor dVIN ○ favor no-VIN ○ no-VIN

COMMENTS

SLIDE 10

p53-IHC PATTERN

○ Basal to para-basal/diffuse overexpression

○ Basal overexpression

○ Null-pattern

○ Cytoplasmic

○ Wild-type

DIAGNOSIS

- ○ dVIN ○ favor dVIN ○ favor no-VIN ○ no-VIN

COMMENTS

SLIDE 11

p53-IHC PATTERN

- ○ Basal to para-basal/diffuse overexpression
- ○ Basal overexpression
- ○ Null-pattern
- ○ Cytoplasmic
- ○ Wild-type

DIAGNOSIS

- ○ dVIN ○ favor dVIN ○ favor no-VIN ○ no-VIN

COMMENTS

SLIDE 12

p53-IHC PATTERN

- ○ Basal to para-basal/diffuse overexpression
- ○ Basal overexpression
- ○ Null-pattern
- ○ Cytoplasmic
- ○ Wild-type

DIAGNOSIS

- [ ] dVIN
- [ ] favor dVIN
- [ ] favor no-VIN
- [ ] no-VIN

COMMENTS

SLIDE 13

p53-IHC PATTERN

- [ ] Basal to para-basal/diffuse overexpression
- [ ] Basal overexpression
- [ ] Null-pattern
- [ ] Cytoplasmic
- [ ] Wild-type

DIAGNOSIS

- [ ] dVIN
- [ ] favor dVIN
- [ ] favor no-VIN
- [ ] no-VIN

COMMENTS

SLIDE 14

p53-IHC PATTERN

- [ ] Basal to para-basal/diffuse overexpression
- [ ] Basal overexpression
- [ ] Null-pattern
- [ ] Cytoplasmic
- [ ] Wild-type

DIAGNOSIS

- ◯ dVIN
- ◯ favor dVIN
- ◯ favor no-VIN
- ◯ no-VIN

COMMENTS

SLIDE 15

p53-IHC PATTERN

- ◯ Basal to para-basal/diffuse overexpression
- ◯ Basal overexpression
- ◯ Null-pattern
- ◯ Cytoplasmic
- ◯ Wild-type

DIAGNOSIS

- ◯ dVIN
- ◯ favor dVIN
- ◯ favor no-VIN
- ◯ no-VIN

COMMENTS

SLIDE 16

p53-IHC PATTERN

- ◯ Basal to para-basal/diffuse overexpression
- ◯ Basal overexpression
- ◯ Null-pattern
- ◯ Cytoplasmic
- ◯ Wild-type

DIAGNOSIS

- ◯ dVIN ◯ favor dVIN ◯ favor no-VIN ◯ no-VIN

COMMENTS

SLIDE 17

p53-IHC PATTERN

- ◯ Basal to para-basal/diffuse overexpression
- ◯ Basal overexpression
- ◯ Null-pattern
- ◯ Cytoplasmic
- ◯ Wild-type

DIAGNOSIS

- ◯ dVIN ◯ favor dVIN ◯ favor no-VIN ◯ no-VIN

COMMENTS

SLIDE 18

p53-IHC PATTERN

- ◯ Basal to para-basal/diffuse overexpression
- ◯ Basal overexpression
- ◯ Null-pattern
- ◯ Cytoplasmic
- ◯ Wild-type

DIAGNOSIS

- ○ dVIN ○ favor dVIN ○ favor no-VIN ○ no-VIN

COMMENTS

SLIDE 19

p53-IHC PATTERN

- ○ Basal to para-basal/diffuse overexpression
- ○ Basal overexpression
- ○ Null-pattern
- ○ Cytoplasmic
- ○ Wild-type

DIAGNOSIS

- ○ dVIN ○ favor dVIN ○ favor no-VIN ○ no-VIN

COMMENTS

SLIDE 20

p53-IHC PATTERN

- ○ Basal to para-basal/diffuse overexpression
- ○ Basal overexpression
- ○ Null-pattern
- ○ Cytoplasmic
- ○ Wild-type

DIAGNOSIS

- ○ dVIN
- ○ favor dVIN
- ○ favor no-VIN
- ○ no-VIN

COMMENTS

SLIDE 21

p53-IHC PATTERN

- ○ Basal to para-basal/diffuse overexpression
- ○ Basal overexpression
- ○ Null-pattern
- ○ Cytoplasmic
- ○ Wild-type

DIAGNOSIS

- ○ dVIN
- ○ favor dVIN
- ○ favor no-VIN
- ○ no-VIN

COMMENTS

SLIDE 22

p53-IHC PATTERN

- ○ Basal to para-basal/diffuse overexpression
- ○ Basal overexpression
- ○ Null-pattern
- ○ Cytoplasmic
- ○ Wild-type

DIAGNOSIS

◯ dVIN ◯ favor dVIN ◯ favor no-VIN ◯ no-VIN

COMMENTS

SLIDE 23

p53-IHC PATTERN

◯ Basal to para-basal/diffuse overexpression

◯ Basal overexpression

◯ Null-pattern

◯ Cytoplasmic

◯ Wild-type

DIAGNOSIS

◯ dVIN ◯ favor dVIN ◯ favor no-VIN ◯ no-VIN

COMMENTS

SLIDE 24

p53-IHC PATTERN

◯ Basal to para-basal/diffuse overexpression

◯ Basal overexpression

◯ Null-pattern

◯ Cytoplasmic

◯ Wild-type

DIAGNOSIS

- ◯ dVIN
- ◯ favor dVIN
- ◯ favor no-VIN
- ◯ no-VIN

COMMENTS

SLIDE 25

p53-IHC PATTERN

- ◯ Basal to para-basal/diffuse overexpression
- ◯ Basal overexpression
- ◯ Null-pattern
- ◯ Cytoplasmic
- ◯ Wild-type

DIAGNOSIS

- ◯ dVIN
- ◯ favor dVIN
- ◯ favor no-VIN
- ◯ no-VIN

COMMENTS

SLIDE 26

p53-IHC PATTERN

- ◯ Basal to para-basal/diffuse overexpression
- ◯ Basal overexpression
- ◯ Null-pattern
- ◯ Cytoplasmic
- ◯ Wild-type

DIAGNOSIS

- ◯ dVIN
- ◯ favor dVIN
- ◯ favor no-VIN
- ◯ no-VIN

COMMENTS

SLIDE 27

p53-IHC PATTERN

- ◯ Basal to para-basal/diffuse overexpression
- ◯ Basal overexpression
- ◯ Null-pattern
- ◯ Cytoplasmic
- ◯ Wild-type

DIAGNOSIS

- ◯ dVIN
- ◯ favor dVIN
- ◯ favor no-VIN
- ◯ no-VIN

COMMENTS

SLIDE 28

p53-IHC PATTERN

- ◯ Basal to para-basal/diffuse overexpression
- ◯ Basal overexpression
- ◯ Null-pattern
- ◯ Cytoplasmic
- ◯ Wild-type

DIAGNOSIS

- ○ dVIN
- ○ favor dVIN
- ○ favor no-VIN
- ○ no-VIN

COMMENTS

SLIDE 29

p53-IHC PATTERN

- ○ Basal to para-basal/diffuse overexpression
- ○ Basal overexpression
- ○ Null-pattern
- ○ Cytoplasmic
- ○ Wild-type

DIAGNOSIS

- ○ dVIN
- ○ favor dVIN
- ○ favor no-VIN
- ○ no-VIN

COMMENTS

SLIDE 30

p53-IHC PATTERN

- ○ Basal to para-basal/diffuse overexpression
- ○ Basal overexpression
- ○ Null-pattern
- ○ Cytoplasmic
- ○ Wild-type

DIAGNOSIS

- ◯ dVIN
- ◯ favor dVIN
- ◯ favor no-VIN
- ◯ no-VIN

COMMENTS

SLIDE 31

p53-IHC PATTERN

- ◯ Basal to para-basal/diffuse overexpression
- ◯ Basal overexpression
- ◯ Null-pattern
- ◯ Cytoplasmic
- ◯ Wild-type

DIAGNOSIS

- ◯ dVIN
- ◯ favor dVIN
- ◯ favor no-VIN
- ◯ no-VIN

COMMENTS

SLIDE 32

p53-IHC PATTERN

- ◯ Basal to para-basal/diffuse overexpression
- ◯ Basal overexpression
- ◯ Null-pattern
- ◯ Cytoplasmic
- ◯ Wild-type

DIAGNOSIS

- ◯ dVIN ◯ favor dVIN ◯ favor no-VIN ◯ no-VIN

COMMENTS

SLIDE 33

p53-IHC PATTERN

- ◯ Basal to para-basal/diffuse overexpression
- ◯ Basal overexpression
- ◯ Null-pattern
- ◯ Cytoplasmic
- ◯ Wild-type

DIAGNOSIS

- ◯ dVIN ◯ favor dVIN ◯ favor no-VIN ◯ no-VIN

COMMENTS

SLIDE 34

p53-IHC PATTERN

- ◯ Basal to para-basal/diffuse overexpression
- ◯ Basal overexpression
- ◯ Null-pattern
- ◯ Cytoplasmic
- ◯ Wild-type

DIAGNOSIS

- ◯ dVIN ◯ favor dVIN ◯ favor no-VIN ◯ no-VIN

COMMENTS

SLIDE 35

p53-IHC PATTERN

- ◯ Basal to para-basal/diffuse overexpression
- ◯ Basal overexpression
- ◯ Null-pattern
- ◯ Cytoplasmic
- ◯ Wild-type

DIAGNOSIS

- ◯ dVIN ◯ favor dVIN ◯ favor no-VIN ◯ no-VIN

COMMENTS

SLIDE 36

p53-IHC PATTERN

- ◯ Basal to para-basal/diffuse overexpression
- ◯ Basal overexpression
- ◯ Null-pattern
- ◯ Cytoplasmic
- ◯ Wild-type

DIAGNOSIS

○ dVIN ○ favor dVIN ○ favor no-VIN ○ no-VIN

COMMENTS

SLIDE 37

p53-IHC PATTERN

○ Basal to para-basal/diffuse overexpression

○ Basal overexpression

○ Null-pattern

○ Cytoplasmic

○ Wild-type

DIAGNOSIS

○ dVIN ○ favor dVIN ○ favor no-VIN ○ no-VIN

COMMENTS

SLIDE 38

p53-IHC PATTERN

○ Basal to para-basal/diffuse overexpression

○ Basal overexpression

○ Null-pattern

○ Cytoplasmic

○ Wild-type

DIAGNOSIS

- ◯ dVIN ◯ favor dVIN ◯ favor no-VIN ◯ no-VIN

COMMENTS

SLIDE 39

p53-IHC PATTERN

- ◯ Basal to para-basal/diffuse overexpression
- ◯ Basal overexpression
- ◯ Null-pattern
- ◯ Cytoplasmic
- ◯ Wild-type

DIAGNOSIS

- ◯ dVIN ◯ favor dVIN ◯ favor no-VIN ◯ no-VIN

COMMENTS

SLIDE 40

p53-IHC PATTERN

- ◯ Basal to para-basal/diffuse overexpression
- ◯ Basal overexpression
- ◯ Null-pattern
- ◯ Cytoplasmic
- ◯ Wild-type

DIAGNOSIS

- ○ dVIN ○ favor dVIN ○ favor no-VIN ○ no-VIN

COMMENTS

SLIDE 41

p53-IHC PATTERN

- ○ Basal to para-basal/diffuse overexpression
- ○ Basal overexpression
- ○ Null-pattern
- ○ Cytoplasmic
- ○ Wild-type

DIAGNOSIS

- ○ dVIN ○ favor dVIN ○ favor no-VIN ○ no-VIN

COMMENTS

SLIDE 42

p53-IHC PATTERN

- ○ Basal to para-basal/diffuse overexpression
- ○ Basal overexpression
- ○ Null-pattern
- ○ Cytoplasmic
- ○ Wild-type

DIAGNOSIS

○ dVIN ○ favor dVIN ○ favor no-VIN ○ no-VIN

COMMENTS

SLIDE 43

p53-IHC PATTERN

○ Basal to para-basal/diffuse overexpression

○ Basal overexpression

○ Null-pattern

○ Cytoplasmic

○ Wild-type

DIAGNOSIS

○ dVIN ○ favor dVIN ○ favor no-VIN ○ no-VIN

COMMENTS

SLIDE 44

p53-IHC PATTERN

○ Basal to para-basal/diffuse overexpression

○ Basal overexpression

○ Null-pattern

○ Cytoplasmic

○ Wild-type

DIAGNOSIS

○ dVIN ○ favor dVIN ○ favor no-VIN ○ no-VIN

COMMENTS

SLIDE 45

p53-IHC PATTERN

○ Basal to para-basal/diffuse overexpression

○ Basal overexpression

○ Null-pattern

○ Cytoplasmic

○ Wild-type

DIAGNOSIS

○ dVIN ○ favor dVIN ○ favor no-VIN ○ no-VIN

COMMENTS

SLIDE 46

p53-IHC PATTERN

○ Basal to para-basal/diffuse overexpression

○ Basal overexpression

○ Null-pattern

○ Cytoplasmic

○ Wild-type

DIAGNOSIS

- ○ dVIN
- ○ favor dVIN
- ○ favor no-VIN
- ○ no-VIN

COMMENTS

SLIDE 47

p53-IHC PATTERN

- ○ Basal to para-basal/diffuse overexpression
- ○ Basal overexpression
- ○ Null-pattern
- ○ Cytoplasmic
- ○ Wild-type

DIAGNOSIS

- ○ dVIN
- ○ favor dVIN
- ○ favor no-VIN
- ○ no-VIN

COMMENTS

SLIDE 48

p53-IHC PATTERN

- ○ Basal to para-basal/diffuse overexpression
- ○ Basal overexpression
- ○ Null-pattern
- ○ Cytoplasmic
- ○ Wild-type

DIAGNOSIS

- dVIN
- favor dVIN
- favor no-VIN
- no-VIN

COMMENTS

SLIDE 49

p53-IHC PATTERN

- Basal to para-basal/diffuse overexpression
- Basal overexpression
- Null-pattern
- Cytoplasmic
- Wild-type

DIAGNOSIS

- dVIN
- favor dVIN
- favor no-VIN
- no-VIN

COMMENTS

SLIDE 50

p53-IHC PATTERN

- Basal to para-basal/diffuse overexpression
- Basal overexpression
- Null-pattern
- Cytoplasmic
- Wild-type

DIAGNOSIS

○ **dVIN** ○ **favor dVIN** ○ **favor no-VIN** ○ **no-VIN**

COMMENTS

-----------------------------------***OUR SINCERE THANKS FOR YOUR PARTICIPATION***--

Instructions for downloading the Nanozoomer app for viewing the whole slide images

1. Please click on the following link to download the Nanozoomer viewer app (NDP.view2).
   https://www.hamamatsu.com/eu/en/product/type/U12388-01/index.html
2. Select the app version appropriate for the operating system of your computer.

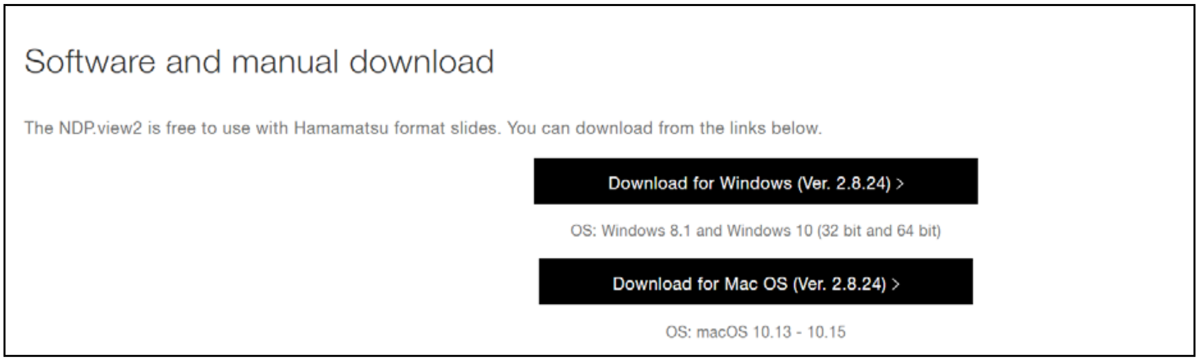

3. The software will be downloaded in a zip file.
4. Click on the zip file and then on the .exe file.

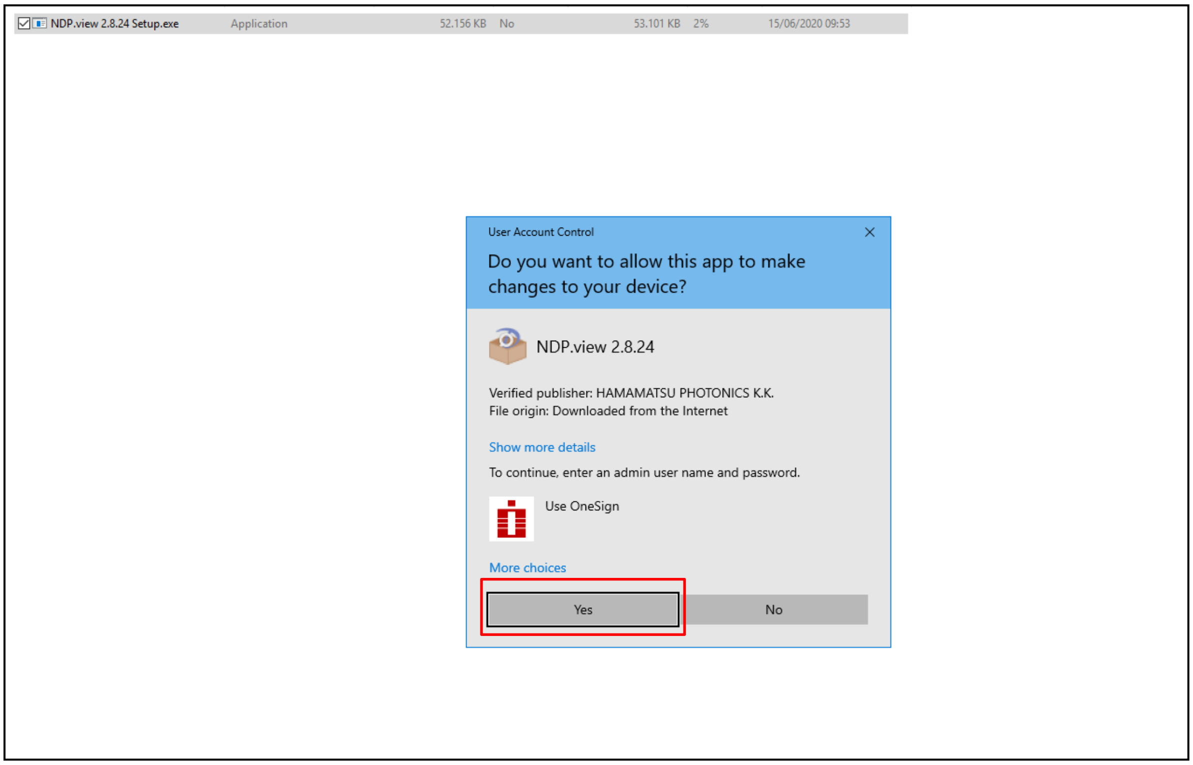

5. This will launch the installation wizard – please select ‘Install’.

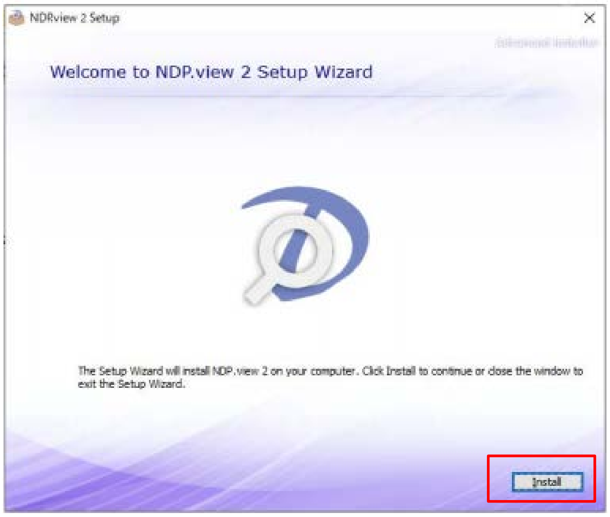

6. The following window will appear when the installation finishes – please select ‘run NDP.view2’ to open the app for viewing the slides, or ‘close’ to complete the install.

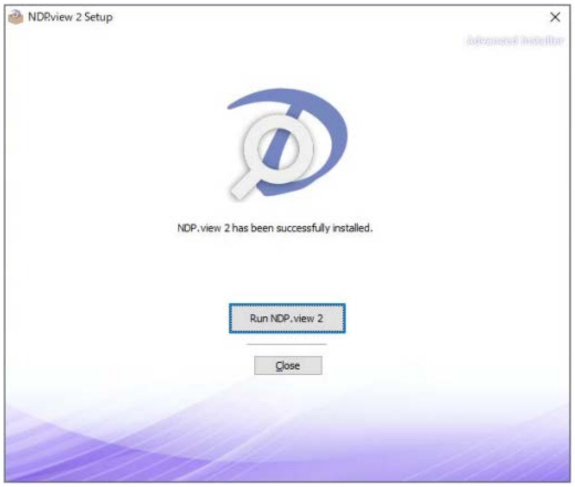

==

Scoring guide: p53 immunohistochemistry

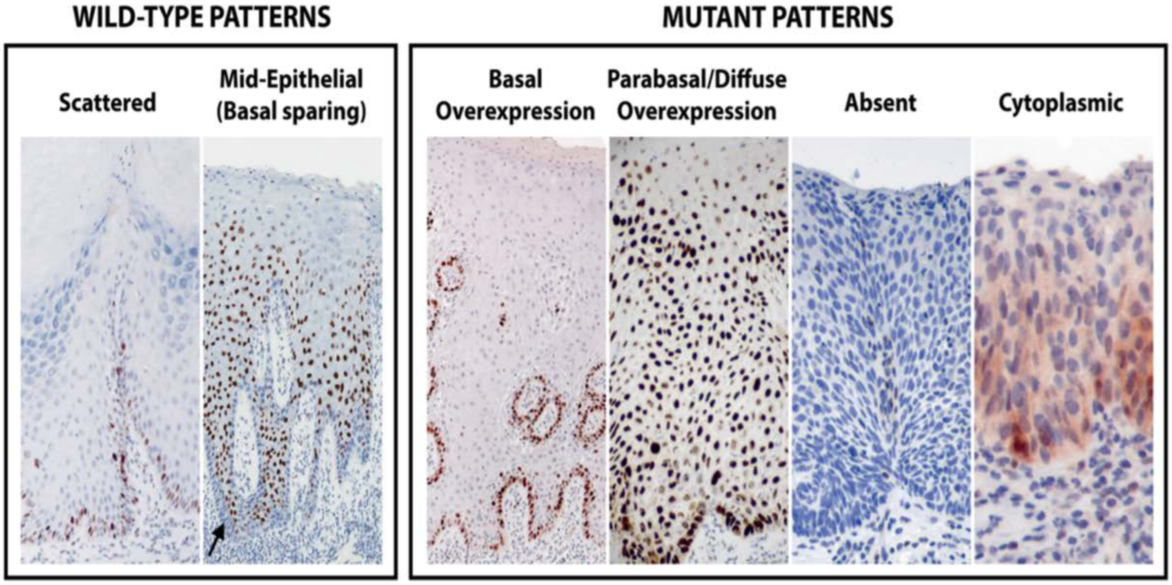

Source: Tessier-Cloutier B, Kortekaas KE, Thompson E, et al. ***Major p53 immunohistochemical patterns in in situ and invasive squamous cell carcinomas of the vulva and correlation with TP53 mutation status.*** Mod Pathol. 2020 Aug;33(8):1595-1605. doi: 10.1038/s41379-020-0524-1. PMID: 32203095.

Description of the p53-expression patterns

Wild-type patterns

Scattered: Heterogeneous nuclear staining of variable intensities in the basal and para-basal layers.
This can be seen in differentiated vulvar intraepithelial neoplasia (dVIN), as well as, in non-dysplastic lesions.

Mid-epithelial (basal sparing): Heterogeneous strong staining in mid-epithelial cells, with notable sparing of basal cells (and sometimes sparing of lower parabasal cells as well).
This is primarily seen in HPV-related high grade squamous intraepithelial lesion (HSIL) and HPV-related vulvar squamous cell carcinoma.

Mutant patterns

Basal overexpression: Uniformly strong nuclear staining in at least 80% of the basal cells without significant parabasal staining.

Parabasal/diffuse overexpression: Uniformly strong nuclear staining of at least 80% of the basal cells and strong parabasal extension.

Absent/Null: Complete absence of staining in the presence of a positive internal control (positive staining in adjacent inflammatory and stromal cells).

Cytoplasmic: Cytoplasmic staining with or without nuclear staining in the presence of a positive internal control (nuclear staining in adjacent inflammatory or stromal cells).

Mutant patterns of p53 have been reported to correlate strongly with the presence of underlying *TP53* mutations, and therefore can be used to support a histological diagnosis of dVIN (1, 2). However, it should also be taken into account that 17 – 42% of dVIN can show a wild-type pattern on p53-IHC (3).

References

1. Kortekaas KE, Solleveld-Westerink N, Tessier-Cloutier B, et al.: Performance of the Pattern Based Interpretation of p53 Immunohistochemistry as a Surrogate for TP53 Mutations in Vulvar Squamous Cell Carcinoma. Histopathology2020.
2. Tessier-Cloutier B, Kortekaas KE, Thompson E, et al.: Major p53 immunohistochemical patterns in in situ and invasive squamous cell carcinomas of the vulva and correlation with TP53 mutation status. Mod Pathol 33: 1595-1605, 2020.
3. Heller DS, Day T, Allbritton JI, et al.: Diagnostic Criteria for Differentiated Vulvar Intraepithelial Neoplasia and Vulvar Aberrant Maturation. J Low Genit Tract Dis2020.
